# Efficient generation of epigenetic disease model mice by epigenome editing using the *piggyBac* transposon system

**DOI:** 10.1186/s13072-022-00474-3

**Published:** 2022-12-16

**Authors:** Takuro Horii, Sumiyo Morita, Mika Kimura, Izuho Hatada

**Affiliations:** 1 Laboratory of Genome Science, Biosignal Genome Resource Center, Institute for Molecular and Cellular Regulation, Gunma University, 3-39-15 Showa-Machi, Maebashi, Gunma 371-8512 Japan; 2 Viral Vector Core, Gunma University Initiative for Advanced Research (GIAR), 3-39-22 Showa-Machi, Maebashi, Gunma 371-8511 Japan

**Keywords:** dCas9, Demethylation, Epigenome editing, *piggyBac*, Silver–Russell syndrome, SunTag

## Abstract

**Background:**

Epigenome-edited animal models enable direct demonstration of disease causing epigenetic mutations. Transgenic (TG) mice stably expressing epigenome-editing factors exhibit dramatic and stable changes in target epigenome modifications. Successful germline transmission of a transgene from founder mice to offspring will yield a sufficient number of epigenome-edited mice for phenotypic analysis; however, if the epigenetic mutation has a detrimental phenotypic effect, it can become difficult to obtain the next generation of animals. In this case, the phenotype of founder mice must be analyzed directly. Unfortunately, current TG mouse production efficiency (TG founders per pups born) is relatively low, and improvements would increase the versatility of this technology.

**Results:**

In the current study, we describe an approach to generate epigenome-edited TG mice using a combination of both the dCas9–SunTag and *piggyBac* (PB) transposon systems. Using this system, we successfully generated mice with demethylation of the differential methylated region of the *H19* gene (*H19*-DMR), as a model for Silver–Russell syndrome (SRS). SRS is a disorder leading to growth retardation, resulting from low insulin-like growth factor 2 (*IGF2*) gene expression, often caused by epimutations at the *H19-IGF2* locus. Under optimized conditions, the efficiency of TG mice production using the PB system was approximately threefold higher than that using the conventional method. TG mice generated by this system showed demethylation of the targeted DNA region and associated changes in gene expression. In addition, these mice exhibited some features of SRS, including intrauterine and postnatal growth retardation, due to demethylation of *H19*-DMR.

**Conclusions:**

The dCas9–SunTag and PB systems serve as a simple and reliable platform for conducting direct experiments using epigenome-edited founder mice.

**Supplementary Information:**

The online version contains supplementary material available at 10.1186/s13072-022-00474-3.

## Background

DNA methylation, represented by 5-methylcytosine (5mC) modification of cytosine–phosphate–guanine (CpG) residues, is a key epigenetic feature with important roles in gene silencing and genome stability [1–3]. Hypermethylated DNA regions are usually linked to silenced and inactive chromatin, while hypomethylated DNA regions are generally associated with gene expression and open chromatin. DNA methylation is relatively stable but can be changed in response to the environment and aging, leading to epigenetic diseases (e.g., cancer, obesity, diabetes, autism, and imprinting disorders) [4–9]. Progress in DNA sequencing technology has enabled genome-wide analysis of epigenetic modifications, yielding huge numbers of candidate disease causing epigenetic mutations [10–12]. Nevertheless, prior to the development of epigenome editing, there were no tools that could directly indicate which epigenetic changes cause disease. Epigenome editing is a technology used for target-specific alteration of DNA methylation or histone modifications and comprises two modules: an effector module, consisting of an epigenetic modification enzyme, and a specific DNA sequence-binding module, such as zinc finger proteins [13], transcription activator-like effectors [14, 15], and catalytically inactive Cas9 (dCas9), based on the clustered regularly interspaced short palindromic repeat (CRISPR)/CRISPR-associated protein 9 (Cas9) system [16–18]. Epigenome editing allows generation of direct evidence of the role of candidate epigenetic mutations in disease at the cellular level. Furthermore, this technology is applied at the animal level. For example, targeted DNA methylation has been achieved in mice by zygote microinjection using MQ1 DNA methyltransferase [19, 20] or DNA methyltransferase 3a (DNMT3a) [21]. Targeted DNA demethylation of hypermethylated regions to reactivate silenced genes has also been achieved in mice using the ten–eleven translocation (TET) 1 hydroxylase fusion protein [21, 22].

The imprinting disorder Silver–Russell syndrome (SRS) is characterized by severe intrauterine and postnatal growth retardation, caused by reduction of insulin-like growth factor 2 (*IGF2*) gene expression [23–25]. Infants with this condition have low birth weight, and often fail to grow and gain weight at the expected rate. Approximately 40% of patients with SRS have epimutations at the *H19-IGF2* locus [26]. We have previously succeeded in generating SRS model mice, in which the *H19*-differentially methylated region (DMR) was demethylated, using the dCas9–SunTag epigenome-editing system [22]. Specifically, TG mice generated by microinjection of an epigenome-editing expression vector into the pronucleus of zygotes exhibited dramatic changes in the targeted epigenome and stability of the edited epigenome because of stable expression of epigenome-editing factors throughout developmental stages. Using this method, if the transgene is successfully transmitted from founder mice to offspring, an epigenome-edited mouse strain can be established, generating sufficient numbers of epigenome-edited mice for phenotypic analysis. By contrast, if the epigenetic mutation has a detrimental phenotypic effect, especially on the fertility or viability of founder mice, it is difficult to obtain the next generation. In such cases, it is necessary to analyze the phenotype of founder mice directly. Unfortunately, the efficiency of TG mouse production by pronuclear injection of plasmid DNA is relatively low (approximately 10% of pups born are TG founders) [22], and improvements in efficiency would increase the versatility of this technology.

One method to improve TG mice production efficiency is viral transgenesis. Using disarmed lentiviral vectors, around 80% of pups born are TG animals [27]; however, this technique has the disadvantage of a relatively small cargo capacity of 9.5 kb due to the limited amount of DNA that can be packaged within the viral particle. As an epigenome-editing vector includes various components, the total length easily exceeds this capacity; therefore, use of viral transgenesis is not feasible. Other methods to increase TG mouse production efficiency include DNA transposon systems, such as *Sleeping beauty*, *Tol2*, and *piggyBac* (PB) [28–30]. A recent study showed that the hyperactive PB transposase (PBase) (hyPBase) has higher activity in both excision and integration assays than other types of transposases [31]. In addition, PB has the unique property of carrying transgenes up to 100 kb [32]. The transposition-dependent PB gene delivery system is very simple. Only a PBase expression vector and a PB transposon vector, including the gene of interest, flanked by two inverted terminal repeat (ITR) sequences, are introduced into the cells. Subsequently, the PBase generated from the PBase expression vector recognizes and binds to the ITRs at both ends of the PB transposon vector. Consequently, the PBase interacts with several host chromosomal sites containing TTAA sequences, allowing individual transgene integration via TTAA sites, to improve integration rates. In addition, the cytoplasmic injection technique used in PBase technology is less damaging to embryos [33] compared with the pronuclear injection approach used in the conventional TG generation approach, which strongly impacts embryo survival rates due to toxicity [29]. In this study, we attempted to generate epigenetic disease model mice by targeted DNA demethylation using the PB transposon system, which enabled efficient transgene integration and high animal viability.

## Results

### Target selection and epigenome-editing system

**Fig. 1:**
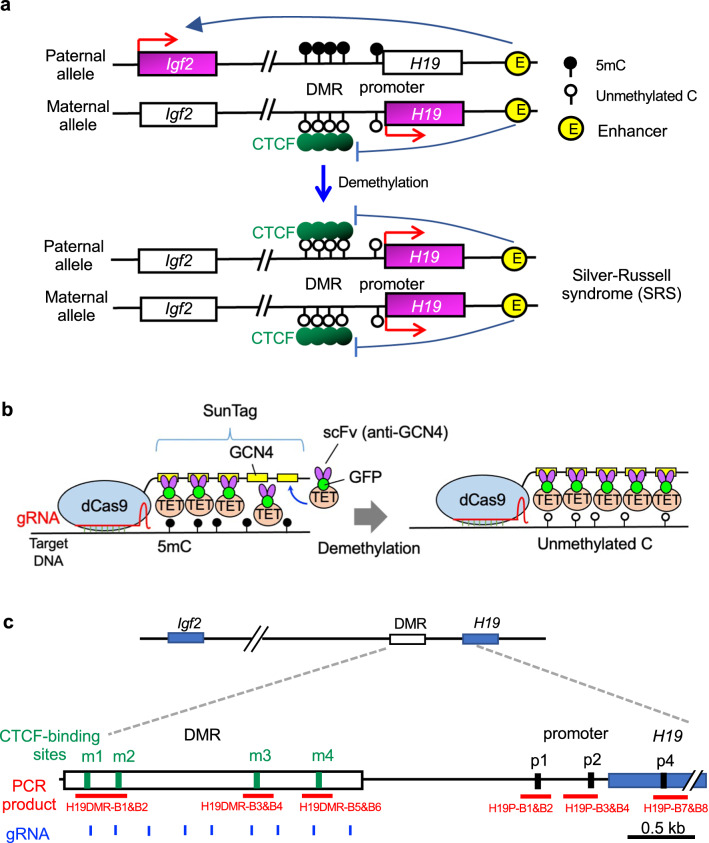
Schematics illustrating targeted DNA demethylation of *H19*-DMR to generate SRS model mice. **a** In mice and humans, *Igf2* is normally expressed from the paternal allele, and *H19* is expressed from the maternal allele. In patients with SRS, DNA demethylation of *H19*-DMR results in biallelic expression of *H19* and repression of *Igf2*. **b** Scheme for CRISPR/Cas9- and SunTag-based induction of demethylation. dCas9 fused to a SunTag (multiple GCN4) can recruit multiple copies of antibody (scFv)-fused TET1CD. Thus, multiple copies of TET1CD hydroxylate specific loci and activate target site-specific demethylation. **c** Schematic of the mouse *H19* locus with four CTCF-binding sites (m1–m4), indicated by green boxes. Locations of gRNA target sites in the *H19*-DMR are indicated by blue bars. PCR amplified regions and names of primers used for DNA methylation analysis are indicated in red. DNA methylation of CpG sites in CTCF-binding sites (m1–m4) and promotor regions (p1–p4) were analyzed

Genomic imprinting is a process, where a gene is differentially expressed depending on whether it has been inherited from the father or mother [36]. The cytosine–phosphate–guanine (CpG) dinucleotides of the *H19*-DMR, which is located between the *Igf2* and *H19* imprinted genes, are normally methylated on the paternal allele and unmethylated on the maternal allele in somatic cells [37] (Fig. 1a, upper). This allele-specific CpG methylation is thought to upregulate and downregulate *Igf2* and *H19* paternal allele expression, respectively. Hypomethylation of paternal *H19*-DMR DNA causes SRS, which is characterized by intrauterine growth retardation due to *Igf2* downregulation (Fig. 1a, lower) [38]. CpG demethylation of the paternal *H19*-DMR allele would be expected to decrease *Igf2* expression and increase *H19* expression through epigenetic regulation of CTCF binding [39]. We previously generated SRS model mice, in which *H19*-DMR was demethylated by epigenome editing. In the current dCas9–SunTag system, SunTag-carrying dCas9 recruits the scFv–sfGFP–TET1CD fusion protein, containing the catalytic domain (CD) of TET1 hydroxylase, to the target locus, leading to efficient targeted DNA demethylation (Fig. 1b) [34]. Nine gRNAs targeting the *H19*-DMR, containing four CTCF-binding sites (m1–m4), were selected to demethylate this region (Fig. 1c).

### Optimization of the PB transposon system for epigenome editing

**Fig. 2:**
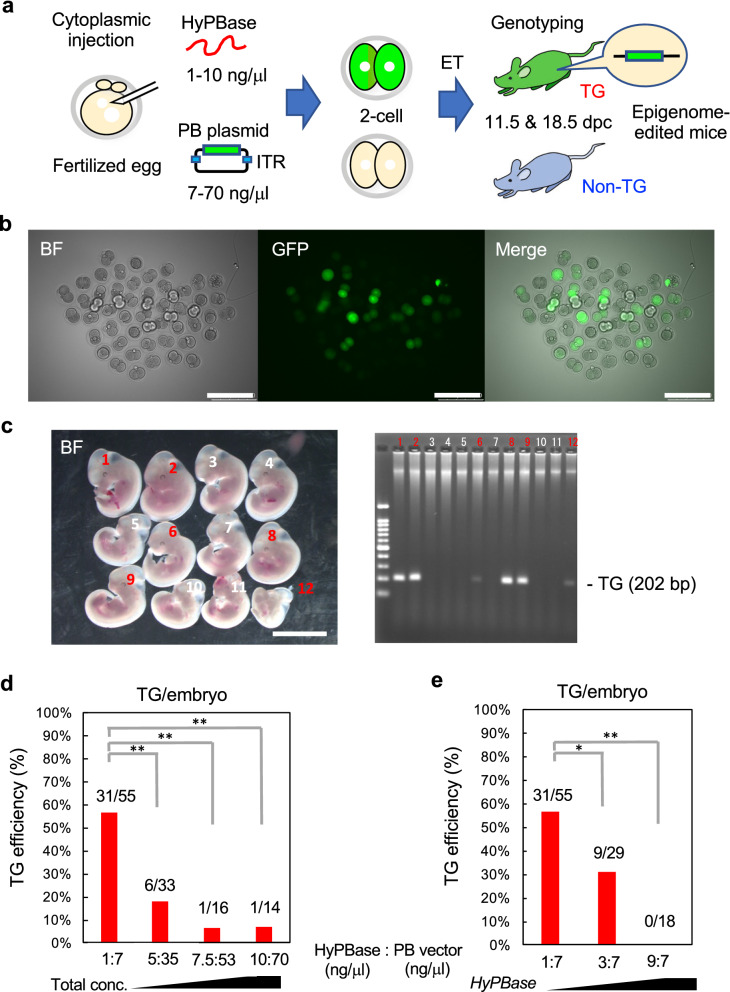
Optimization of hyPBase and PB vector concentrations for generation of TG epigenome-edited mice. **a** Schematic of the generation of epigenome-edited mice by cytoplasmic injection of hyPBase and PB vector into fertilized eggs. **b** Embryos at the 2-cell stage (next day after injection). *BF* bright field. Scale bars, 250 μm. **c** Embryos at 11.5 dpc and the associated agarose gel electrophoresis image of PCR products targeting the dCas9 transgene. Red numbers indicate TG mice in which the PB vector is integrated. Scale bar, 5 mm. **d** TG efficiency at 11.5 dpc under a constant concentration ratio (1:7) of hyPBase to PB vector. **e** TG efficiency at 11.5 dpc under a constant PB vector concentration (7 ng/μL). *P < 0.05, **P < 0.01 (two-tailed Student’s *t* test)

The concentration and ratio of hyPBase transposase and PB epigenome-editing vector can strongly affect TG efficiency [33], especially when the inserted vector is long; therefore, we conducted optimization of the system. A mixture of hyPBase mRNA and PB epigenome-editing vector (17.7 kb, Additional file 1: Fig. S1a) was injected into the cytoplasm of fertilized eggs, and 2-cell stage embryos were transferred into the oviducts of pseudopregnant mice (Fig. 2a). Embryos were then recovered at 11.5 days post coitus (dpc) to confirm the embryo survival rate and TG animal generation efficiency. To determine the optimal concentration, we fixed the molar ratio of hyPBase to PB vector (2.8 to 1) as reported [33] and changed the total weight/volume injected. According to GFP intensity analysis, epigenome-editing factors were indeed expressed in 2-cell stage (Fig. 2b) and 11.5 dpc embryos (Fig. 2c; Additional file 1: Fig. S2). Contrary to expectations, the highest TG efficiency (56.4% TG embryos recovered) was observed with the lowest concentrations of hyPBase and PB vector (1 ng/μL and 7 ng/μL, respectively) (Fig. 2d). Next, to determine the best ratio of hyPBase and PB epigenome-editing vector, we fixed the PB vector concentration at 7 ng/μL and increased the hyPBase concentration; however, TG efficiency did not increase under these conditions (Fig. 2e), indicating that hyPBase is fully functional at 1 ng/μL. Furthermore, no significant difference in embryo survival rate was observed among the various concentration conditions (Additional file 1: Fig. S3). Based on these experiments, we determined that 1 ng/μL of hyPBase and 7 ng/μL of PB vector were optimal and proceeded with the generation of epigenome-edited mice using these conditions.

### Targeted DNA demethylation in *H19*-DMR

**Fig. 3:**
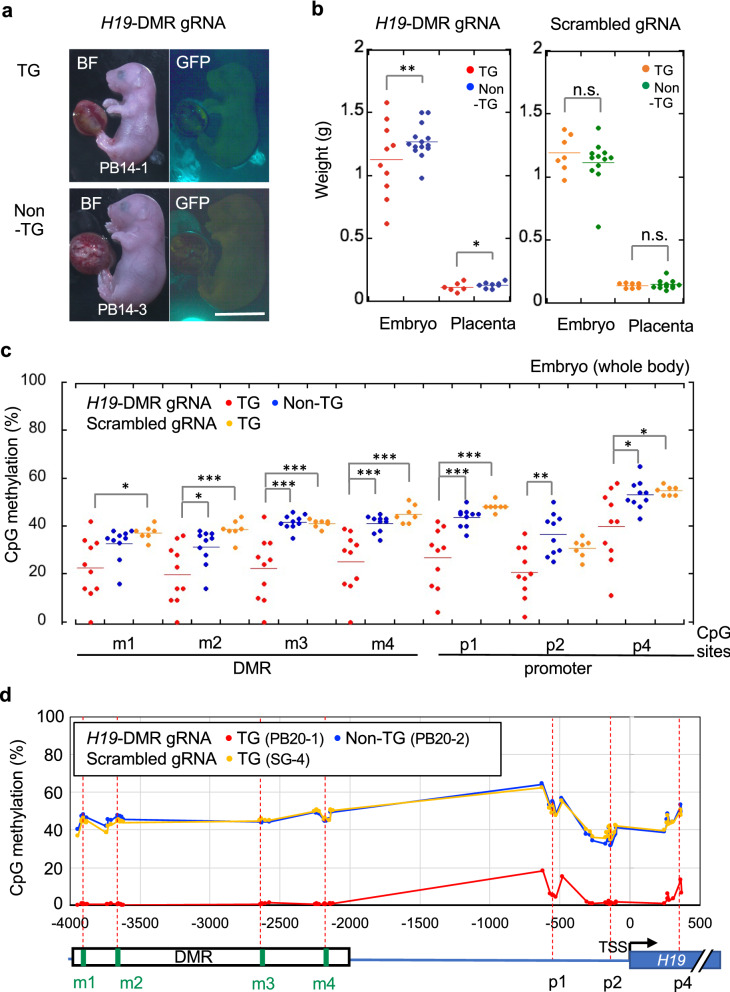
Generation of epigenome-edited TG mice using the optimized PB system. **a** Vector-integrated (TG) embryos (18.5 dpc) were generated using optimized concentrations of hyPBase and PB vector containing *H19*-DMR gRNA. Non-TG:control mice in which the epigenome-editing vector did not successfully integrate. *BF* bright field. Scale bar, 1 cm. **b** Body and placental weights of TG and non-TG mice with *H19*-DMR gRNA or scrambled gRNA. **P < 0.01 (two-tailed Student’s *t* test). **c** Combined bisulfite restriction analysis (COBRA) for *H19*-DMR and promoter regions. Significant demethylation was observed in TG embryos with *H19*-DMR gRNA. *P < 0.05, **P < 0.01, ***P < 0.001 (one-way ANOVA followed by Tukey’s post hoc HSD test). **d** Amplicon bisulfite sequencing analysis targeting 59 CpG sites among three representative samples. *TSS* transcription start site

**Fig. 4:**
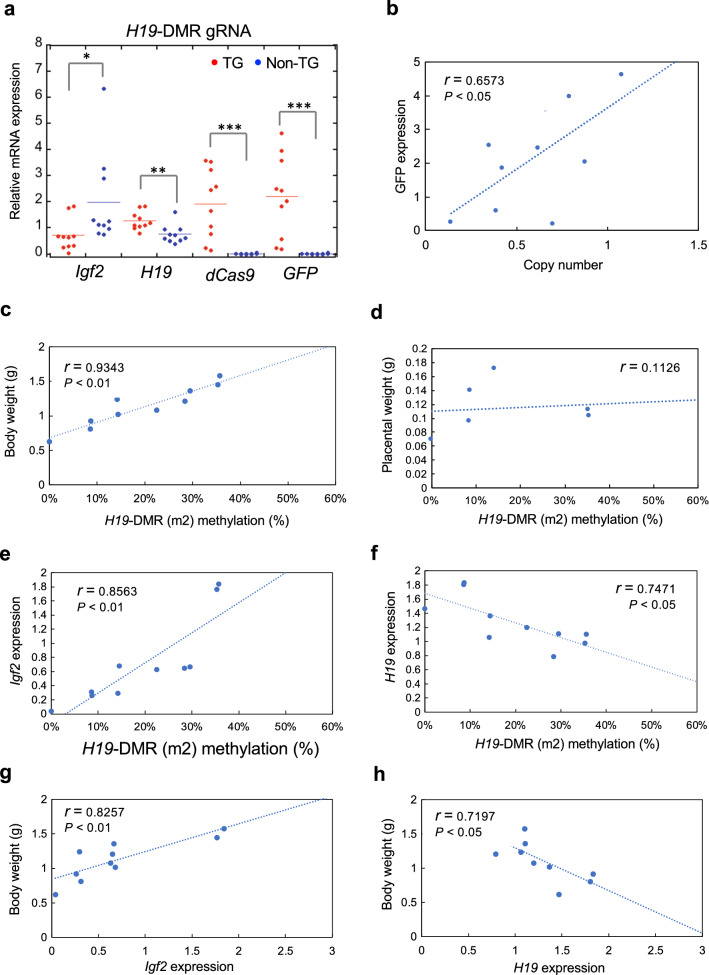
Strong correlations among DNA methylation, gene expression, and body weight in TG epigenome-edited mice at 18.5 dpc. **a** qRT-PCR analysis of mRNA expression. *P < 0.05, **P < 0.01, ***P < 0.001 (two-tailed Student’s *t* test). Correlations among **b** transgene copy number and GFP expression, **c** *H19*-DMR methylation (m2 site) and body weight, **d** *H19*-DMR methylation (m2 site) and placental weight, **e**, **f** *H19*-DMR methylation (m2 site) and gene expression, and **g**, **h** gene expression and body weight, measured by calculating Pearson’s correlation coefficient values (*r*)

TG epigenome-edited mice were generated using the optimized PB system. Two-cell stage embryos were transferred into the oviducts of pseudopregnant mice after injection of hyPBase and PB vector into the cytoplasm of fertilized eggs. Embryos and placentas were recovered at 18.5 dpc, just before birth. In total, 24.8% of transferred embryos survived to 18.5 dpc (Table 1); this survival rate is not significantly different from that achieved using conventional methods based on plasmid pronuclear injection (24.8 vs. 23.6%). By contrast, the TG method based on the PB system showed significantly higher TG production efficiency than the conventional method (37.0 vs. 13.0%, *P* = 0.0218). According to GFP intensity, epigenome-editing factors were indeed expressed in TG mice with *H19*-DMR gRNA (Fig. 3a), which had significantly lower body and placental weight than non-TG control mice without vector integration (Fig. 3b). By contrast, TG mice with scrambled gRNA did not show reduction of either body or placental weight (Fig. 3b). These findings indicate that TG mice with *H19*-DMR gRNA mimic the phenotype of intrauterine growth retardation observed in patients with SRS. Next, DNA methylation status in the *H19*-DMR and promoter regions was examined by combined bisulfite restriction analysis (COBRA). Despite variations in methylation rates, significant DNA demethylation in the *H19*-DMR and promoter regions was observed in TG mice with *H19*-DMR gRNA (Fig. 3c). Furthermore, amplicon bisulfite sequencing analysis targeting 59 CpG sites revealed considerable demethylation throughout the *H19*-DMR and promoter regions in a TG mouse with *H19*-DMR gRNA (Fig. 3d). TG mice showed significant downregulation of *Igf2* and upregulation of *H19* expression (Fig. 4a). In addition, PB vector-derived dCas9 and *GFP* expression levels varied among TG mice (Fig. 4a), and *GFP* expression level was associated with transgene copy number (Fig. 4b). DNA methylation variations in TG mice were strongly correlated with *Igf2*/*H19* expression and body weight (Fig. 4c–h; Additional file 1: Figs. S4 and S5). These data demonstrate that targeted demethylation of *H19*-DMR changes *Igf2/H19* gene expression patterns and induces SRS-like phenotypes, including reduced body weight.

**Table 1:** Efficiency of TG mouse production by PB and conventional methods

Transgenic method	Number of ET	Number of embryos (18.5 dpc)	Number of TG	Embryos/ET (%)	TG/embryos (%)
PB	109	27	10	24.8%	37.0%
Conventional	195	46	6	23.6%	13.0%
*P* value				0.8888	0.0218*

*ET* embryos transferred

*P < 0.05

**Fig. 5:**
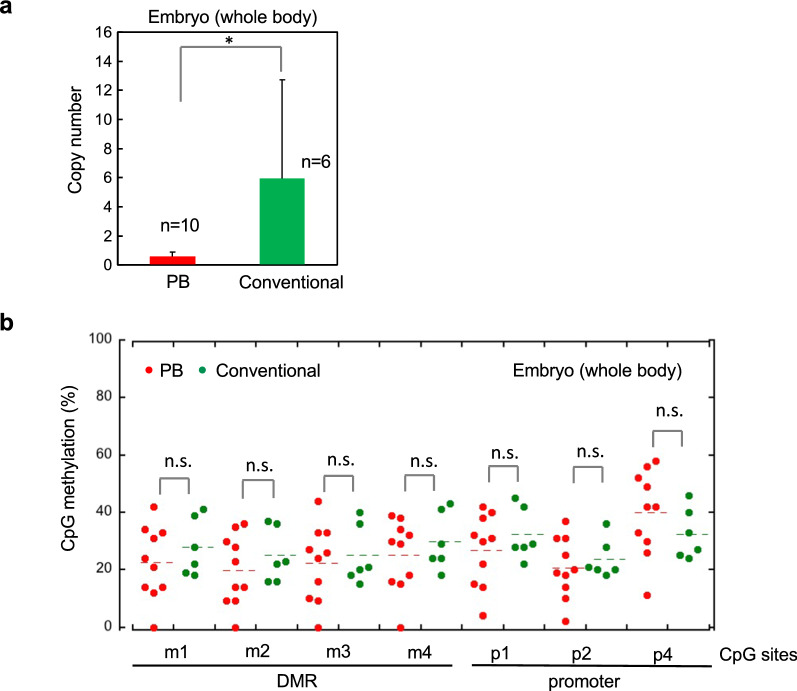
Transgene copy number and DNA methylation levels of TG mice generated by PB and conventional methods. **a** Transgene copy number in TG mouse embryos (18.5 dpc) generated using the PB system is lower than that produced using a conventional approach. **b** No significant differences were observed between *H19*-DMR and promoter region methylation in TG mouse embryos generated by PB and conventional methods. The CpG methylation data of TG mice generated by conventional methods were adopted from our previous report [22]. Error bars, mean ± s.d. *P < 0.05, n.s., not significant (two-tailed Student’s *t* test)

Next, an integration site was identified in each of five TG mice. Four TG mice had transgene integration via TTAA sites, while the other presumably integrated via non-homologous end-joining (Additional file 1: Fig. S6). Then, we compared the copy numbers of integrated transgenes generated using the PB and conventional TG methods. TG mice generated using the PB system had a lower transgene copy number than those generated by pronuclear injection (Fig. 5a). Despite the differences in copy number, TG mice generated using the PB system showed sufficient demethylation of the *H19*-DMR and promoter regions, and there was no significant difference in the CpG methylation levels in these regions between mice generated by PB and conventional approaches (Fig. 5b).

### SRS-like phenotype in TG epigenome-edited mice

**Fig. 6:**
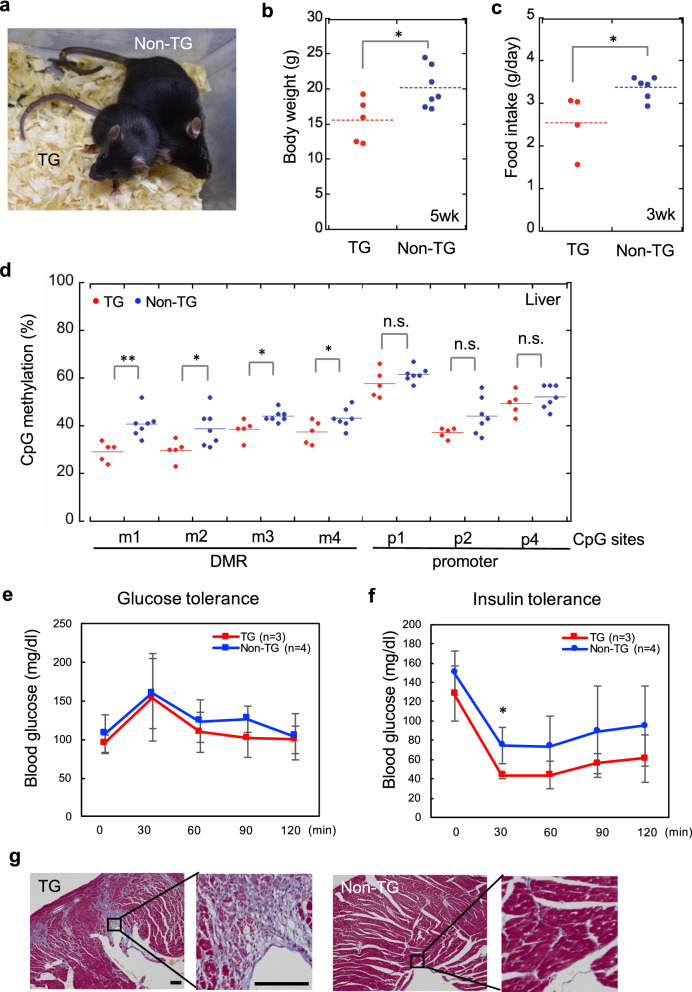
Postnatal development and phenotype of SRS model mice. **a** Appearance of TG and Non-TG mice at 5 weeks. **b** Growth retardation of male TG mice continued to be observed at 5 weeks. **c** Food intake (g/day) was examined in male TG and Non-TG mice (3 weeks). **d** *H19*-DMR methylation status in the liver at 5 weeks analyzed by COBRA. TG mice showed frequent demethylation. **e** Glucose tolerance and **f** insulin tolerance tests in TG and Non-TG male mice (4 and 5 weeks, respectively) fed normal chow were performed after an overnight fast. Blood glucose concentration tended to be lower in TG mice. **g** TG mice (2/3) showed fibrosis in cardiac muscle fiber by Masson's trichrome stain, whereas this phenotype was not observed in non-TG mice (0/3). Scale bar, 100 μm. Error bars, mean ± s.d. *P < 0.05, **P < 0.01, *n.s.* not significant (two-tailed Student’s *t* test)

SRS-like postnatal phenotypes were compared between TG and Non-TG mice. Similar to patients with SRS, weight gain after birth was severely restricted in epigenome-edited TG mice (Fig. 6a, b). Low food intake by TG mice may be a factor influencing growth retardation after birth (Fig. 6c; Additional file 1: Fig. S7). According to methylation analysis of liver samples, *H19*-DMR hypomethylation continued to be observed in TG mice at 5 weeks (Fig. 6d); however, the difference in methylation levels was smaller than that observed at 18.5 dpc. Epigenome-edited mice tended to be hypoglycemic (Fig. 6e, f) and exhibited muscle fiber degeneration/fibrosis in the cardiac muscle, as previously observed [22] (Fig. 6g).

## Discussion

According to our previous study, TG mice, which stably express epigenome-editing factors throughout the body, are a practical way to generate epigenome-edited mice [22]; however, the conventional technique of generating TG mice, in which linearized plasmids are injected into the pronucleus of fertilized eggs, is relatively inefficient. In particular, the conventional TG method is unsuitable for analysis using founder TG mice, because large numbers of TG mice cannot be generated simultaneously. In this study, we applied a PB transposon system to generate TG epigenome-edited mice, which increased the production efficiency of TG mice by approximately threefold compared to the conventional method (PB:conventional = 37.0%:13.0%). Furthermore, the TG mice obtained by the PB system showed sufficient demethylation of the *H19*-DMR, changes in *Igf2* and *H19* expression, and reduced birth weight. The strong correlations among DNA methylation rate, *Igf2/H19* expression level, and body weight provide direct evidence that DNA demethylation of the *H19*-DMR induces intrauterine growth retardation, as observed in patients with SRS. In addition to intrauterine growth retardation, the present SRS model mice showed other characteristics, including poor postnatal growth, reduced food intake, fibrosis of the cardiac muscle, and abnormal blood glucose levels, as previously reported [22].

In the PB system, one transgene copy is theoretically inserted per TTAA site; therefore, numerous copy numbers were expected to integrate in multiple genome sites; however, TG mice generated by the PB method showed lower copy numbers of the inserted transgene than in those produced conventionally. The detailed reason for this finding is currently unknown, but it is noteworthy that transgene copy number is predominantly ‘one’ when transgene length exceeds 10 kb [32]. Although the cell types and methods used to introduce the PB system were different, the same results could have been obtained in the current study using a vector of approximately 17 kb. Such a reduction of transgene copy number may be expected to negatively affect epigenome-editing efficiency; however, comparable DNA demethylation rates were found in mice generated using the PB and conventional methods (Fig. 5). Transgene expression is affected by copy number and by position effects, DNA methylation, histone modifications, and other epigenetic factors [40–42]. Transgenes are often susceptible to gene silencing, and high copy number transgenes are more likely to be silenced [42]. Furthermore, PB insertions are associated with expressed genes and markers of open chromatin structure, and are excluded from heterochromatin [43]. Our results indicate that transgene copy number is not necessarily critical for DNA demethylation by the PB epigenome-editing system.

Here, we have proposed a very simple and reliable system for epigenome editing. To obtain epigenome-edited mice, researchers only need to introduce hyPBase mRNA and a PB transposon vector, including epigenome-editing factors, to fertilized eggs. Once newborn mice are obtained, the success of epigenome editing can be easily visualized by the expression of GFP, prior to detailed epigenetic analysis. We hope that this method, which combines the efficiency, stability, and simplicity for epigenome editing, will develop into a standard method for producing epigenome-edited animals in the future.

## Conclusions

A combination of the dCas9–SunTag and PB systems can be used to obtain epigenome-edited mice more efficiently than conventional methods, supplying sufficient numbers of epigenome-edited mice for phenotypic analysis of founder mice in a single experiment. This system will open the door to a wide range of future applications in epigenetic research, as well as clinical medicine, which have previously been challenging because of difficulties in obtaining subsequent mouse generations.

## Methods

### Animals

B6D2F1 mice were purchased from CLEA Japan (Kawasaki, Japan). ICR mice were purchased from Charles River Japan (Yokohama, Japan).

### Vector construction

We previously reported an all-in-one epigenome-editing vector, including dCas9 fused with five copies of GCN4 and an anti-GCN4 peptide antibody (scFv)–sfGFP–TET1CD fusion protein (pPlatTET–gRNA2, Addgene plasmid 82559) [34]. Furthermore, we constructed an all-in-one epigenome-editing vector including nine guide RNAs (gRNAs) targeting *H19*-DMR (pPlatTET–gRNA2–H19DMRx9) [22]. In this study, a PB epigenome-editing vector (pPlatPBTET–gRNA2–H19DMRx9) was constructed from pPlatTET–gRNA2–H19DMRx9, with two ITR sequences derived from pPB-LR5 (Additional file 1: Fig. S1a). For the control experiment, a pPlatPBTET–gRV2 vector containing scrambled gRNA was also constructed (Additional file 1: Fig. S1b).

### In vitro transcription of hyPBase

The T7 promoter was added to the hyPBase coding region by PCR amplification using specific primers (Additional file 2), with pCMV–hyPBase [35] as the template. The amplified hyPBase PCR product was gel purified and used as a template for in vitro transcription with an mMESSAGE mMACHINE T7 ULTRA kit (Invitrogen). HyPBase mRNA was purified using an MEGAclear kit (Invitrogen) and eluted into RNase-free water. RNA sample quality was checked by gel electrophoresis.

### Preparation of embryos

B6D2F1 female mice (8–10 weeks) were induced to superovulate by injecting 7.5 units of pregnant mare’s serum gonadotropin (SEROTROPIN; ASKA Pharmaceutical, Tokyo, Japan), followed 48 h later by 7.5 units of human chorionic gonadotropin (hCG; GONATROPIN, ASKA Pharmaceutical). After administration of hCG, females were mated with B6D2F1 males. Zygotes were isolated from the oviduct 21 h later. After treatment with M2 medium (Sigma-Aldrich, St. Louis, MO, USA) supplemented with 0.1% hyaluronidase (Sigma-Aldrich) for a few minutes, fertilized eggs were washed with M2 medium and then transferred to drops of M16 medium (Sigma-Aldrich), supplemented with penicillin and streptomycin, at 37 °C under 5% CO_2_ in air.

### Microinjection of zygotes

Microinjection was performed at 24–27 h post-hCG injection, as previously reported [22]. In brief, the pPlatPB–TET–gRNA2–H19DMRx9 vector and hyPBase RNA were injected into the cytoplasm of fertilized eggs. Injected embryos were cultured in M16 medium at 37 °C under 5% CO_2_ in air. The next day, embryos that had developed to the 2-cell stage were transferred into the ampulla of the oviduct of pseudopregnant ICR females. For vector integration analysis, genomic DNA was extracted from whole embryos at 11.5 dpc and 18.5 dpc, and tail tips of weanling mice, using a DNA extraction kit (DirectPCR Lysis Reagent, Mouse Tail; Viagenbiotech, CA, USA). PCR analysis was performed using a primer set for *dCas9* (Additional file 2).

### DNA methylation analysis

Genomic DNA was extracted from the whole bodies of 18.5 dpc embryos and the livers of 5-week-old mice by phenol/chloroform extraction. Purified DNA samples (500 ng) were processed using an Epitect Plus DNA Bisulfite Kit (QIAGEN) according to the manufacturer’s instructions. Modified DNA was amplified using TaKaRa Taq (TaKaRa, Kusatsu, Japan) and the PCR primers described in Additional files 2 and 3. Percentages of demethylated CpG sites were determined by COBRA. Briefly, amplified fragments were cleaved with restriction enzymes (Additional files 2 and 3) whose recognition sites were located at the CpG sites, and then separated and quantified using capillary and microchip electrophoresis (MCE-202 MultiNA, Shimadzu, Kyoto, Japan). Methylation levels were calculated as the percentage of cleaved DNA (mV･μm) among total DNA (mV･μm).

### Bisulfite amplicon sequencing analysis

For comprehensive CpG methylation analysis around the *H19* gene, genomic DNA samples from each of three mice (PB20-1, PB20-2, and SG-4) were treated using the Epitect Plus DNA Bisulfite Kit (QIAGEN) and amplified using six primer pairs (Additional file 2). Pooled PCR products were then used for preparation of a fragment library, as previously reported [22]. Prepared libraries were then sequenced on an Illumina NextSeq 500 (Illumina, San Diego, CA). Generated raw sequence data in FASTQ format were imported into CLC Genomics Workbench 22.0.1 (QIAGEN), trimmed using Trim reads 2.6 tool, and mapped to the reference sequence (NC_000073, *Mus musculus* strain C57BL/6J chromosome 7, GRCm38.p6 C57BL/6J) using the Map Reads to Reference tool (version 1.6). For CpG methylation analysis, trimmed libraries were mapped to the reference sequence as described above using the Map Bisulfite Reads to Reference tool, and 5-mC percentages were calculated using the Call Methylation levels tool (version 1.4) (Additional file 4).

### RT-quantitative PCR (qPCR) analysis

Total RNA was isolated from whole bodies of 18.5 dpc embryos using TRIzol reagent (Invitrogen). Isolated RNA (8.5 μL, 2 μg) was treated with DNase I (50 U mL^−1^) in a total volume of 10 μL at 37 °C for 20 min. DNase I was inactivated by adding 0.8 μL of 25 mM EDTA and incubating samples at 75 °C for 10 min. cDNA was produced from each RNA sample, using random primers (Invitrogen, Carlsbad, CA) and SuperScript II (Invitrogen), according to the manufacturer’s instructions. Reverse transcription reactions were diluted tenfold with water before qPCR. Gene expression levels of *Igf2*, *H19*, and *dCas9* were measured using a LightCycler 96 (Roche) and TB Green Premix Ex Taq II (TaKaRa), according to the manufacturer’s instructions. Gene expression levels were normalized against those of *Gapdh*. Primer sequences are described in Additional file 2.

### Copy number analysis

Transgene (*dCas9*) copy number was measured using a LightCycler 96 (Roche) and TB Green *Premix Ex Taq* II (TaKaRa) according to the manufacturer’s instructions. In brief, 10 ng of genomic DNA from 18.5 dpc embryos was amplified by qPCR using *dCas9* and *Dnmt3b* (endogenous gene) primer sets (Additional file 2). A mixture of pPlatPBTET–gRNA2–H19DMRx9 (for *dCas9*) and pCAG–Dnmt3b (for *Dnmt3b*) was used as a copy number control. The *dCas9* copy number for each sample was normalized to that of *Dnmt3b.*

### Determination of vector insertion loci

Inverse PCR [44] was conducted as illustrated in (Additional file 1: Fig. S6a). Genomic DNA (1 µg) extracted from each of 10 18.5 dpc TG mouse embryos was digested with the *Mbo*I restriction enzyme. Self-ligation was induced using a DNA Ligation Kit (TaKaRa) to generate circular DNA molecules. DNA fragments including the PB vector and unknown integration loci were amplified by nested-PCR using two sets of primers (Additional file 2). After agarose gel electrophoresis, PCR products were purified using a MinElute Gel Extraction Kit (QIAGEN) and the unknown integration loci were confirmed by direct sequencing.

### Statistical analysis

The Fisher’s exact probability test was used to compare embryonic development and efficiency of TG mouse production. DNA methylation, mRNA expression, and body and placental weights were analyzed by Student’s *t* test (two-tailed) for pairwise comparisons or by one-way analysis of variance (ANOVA) followed by Tukey’s post hoc HSD test for multiple comparisons. Pearson’s correlation coefficient values (*r*) were calculated to analyze correlations between variables. Data are presented as mean and standard deviation (s.d.). *P* < 0.05 was considered significant. Detailed data, including body weight, placental weight, DNA methylation, and gene expression, in 18.5 dpc embryos are included in Additional file 5.

## Supplementary Information

**Additional file 1: Fig. S1.** Plasmid maps of the all-in-one vectors used in this study. **Fig. S2.** High TG integration efficiency in 11.5 dpc embryos generated using 1 and 7 ng/μl of hyPBase and PB vector, respectively. **Fig. S3.** Optimization of hyPBase and PB vector concentrations for generation of TG epigenome-edited mice. **Fig. S4.** Correlation of embryo DNA methylation with embryo and placental weights. **Fig. S5.** Correlation between H19-DMR DNA methylation at sites m1–m4 and gene expression. **Fig. S6.** Representative chromatograms of transgene integration loci. **Fig. S7.** Correlation between food intake and body weight.

**Additional file 2.** gRNA used in this study. Primers for in vitro transcription. Primers to check for vector integration. Primers for COBRA and amplicon bisulfite sequencing. Primers for quantitative real-time RT-PCR. Primers for copy number analysis. Primers for inverse PCR (nested PCR).

**Additional file 3. ** A detailed locus map, including gRNA, targeting primers, and assayed CpGs.

**Additional file 4.** Source data of amplicon bisulfite sequencing analysis.

**Additional file 5.** Detailed data, including body weight, placental weight, DNA methylation, and gene expression in18.5 dpc embryos.

## Data Availability

The data sets supporting the conclusions of this article are included within the article and its additional files.

## Abbreviations

5mC: 5-Methylcytosine
Cas9: CRISPR-associated protein 9
CD: Catalytic domain
COBRA: Combined bisulfite restriction analysis
CpG: Cytosine–phosphate–guanine
CRISPR: Clustered regularly interspaced short palindromic repeat
dCas9: Catalytically inactive Cas9
DMR: Differentially methylated region
DNMT3a: DNA methyltransferase 3a
dpc: Days post coitus
gRNA: Guide RNA
hyPBase: Hyperactive PB transposase
hCG: Human chorionic gonadotropin
ITR: Inverted terminal repeat
PB: *piggyBac*
qPCR: RT-quantitative PCR
scFv: Anti-GCN4 peptide antibody
s.d.: Standard deviation
SRS: Silver–Russell syndrome
TET: Ten–eleven translocation
TG: Transgenic
