# Supplementary material for: Efficient generation of epigenetic disease model mice by epigenome editing using the piggyBac transposon system
Source: Epigenetics Chromatin. 2022 Dec 16;15:40. doi: 10.1186/s13072-022-00474-3 (PMC9756621; doi:10.1186/s13072-022-00474-3)
Supplement: Supplementary file 1 — Additional file 1: Fig. S1. Plasmid maps of the all-in-one vectors used in this study. Fig. S2. High TG integration efficiency in 11.5 dpc embryos generated using 1 and 7 ng/μl of hyPBase and PB vector, respectively. Fig. S3. Optimization of hyPBase and PB vector concentrations for generation of TG epigenome-edited mice. Fig. S4. Correlation of embryo DNA methylation with embryo and placental weights. Fig. S5. Correlation between H19-DMR DNA methylation at sites m1–m4 and gene expression. Fig. S6. Representative chromatograms of transgene integration loci. Fig. S7. Correlation between food intake and body weight. [file 13072_2022_474_MOESM1_ESM.pdf]

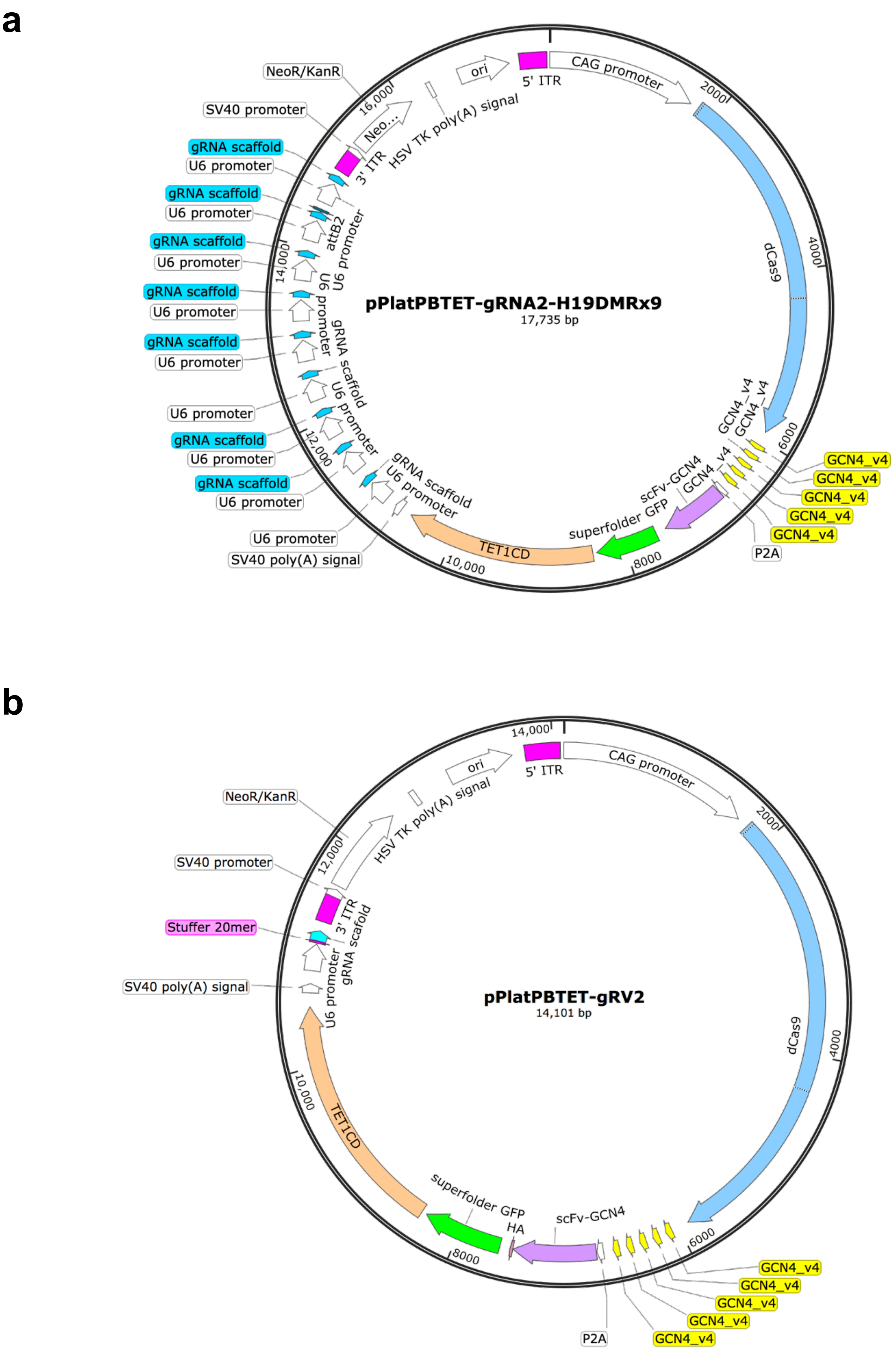

Fig. S1 Plasmid maps of the all-in-one vectors used in this study.
(a) pPlatPBTET-gRNA2-H19DMRx9. **(b)** pPlatPBTET-gRV2 (scrambled gRNA).

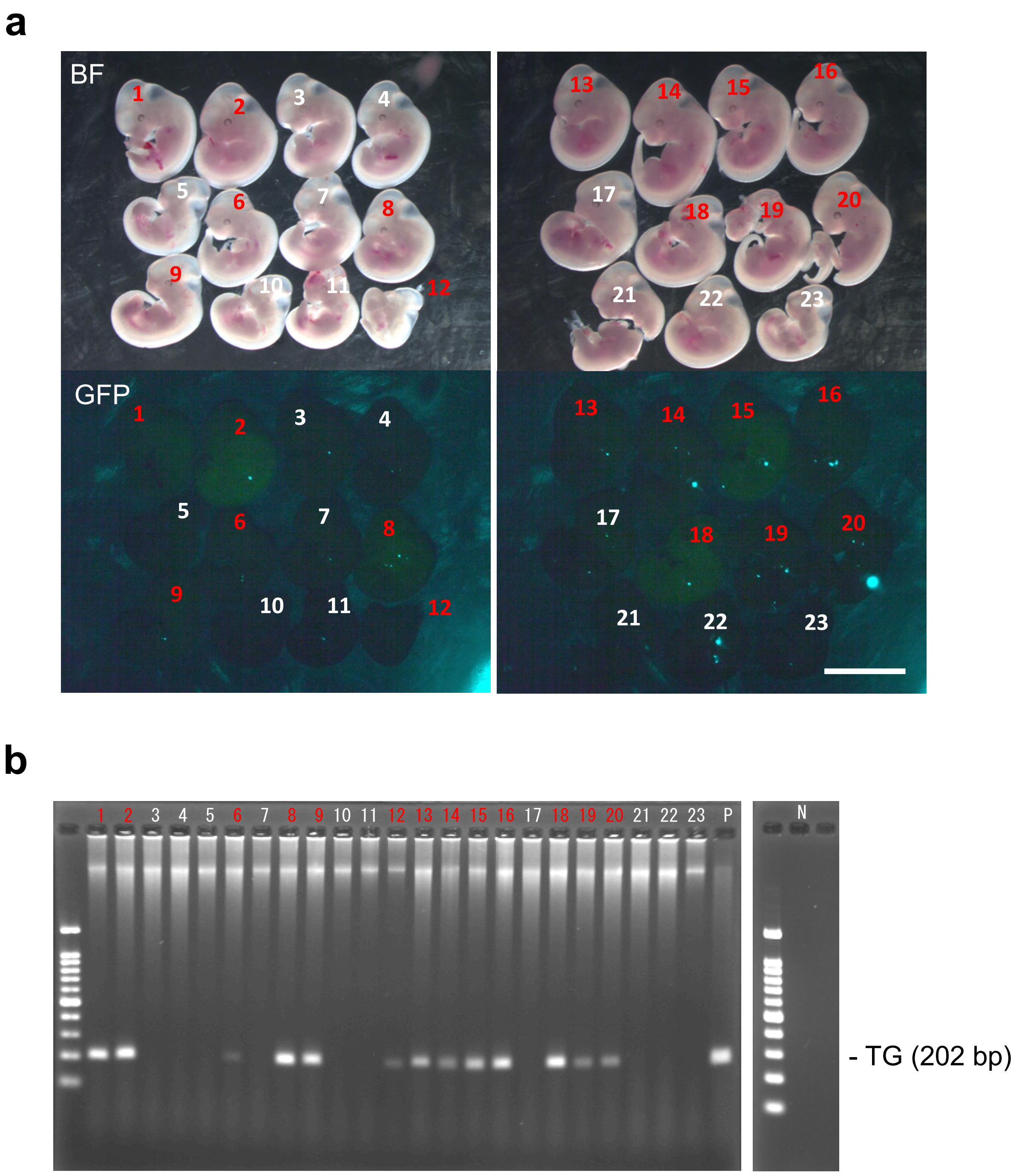

Fig. S2 High TG integration efficiency in 11.5 dpc embryos generated using 1 and 7 ng/µl of hyPBase and PB vector, respectively. **(a)** Representative bright field (BF) and GFP fluorescence images of embryos at 11.5 dpc. Red numbers indicate TG mice in which the PB vector was integrated. Scale bar, 5 mm. **(b)** Agarose gel electrophoresis image of PCR products amplified using the primers dCas9-F and dCas9-R to detect PB vector insertion in the embryos shown in panel 'a'. P, positive control (TG control); N, negative (no template) control.

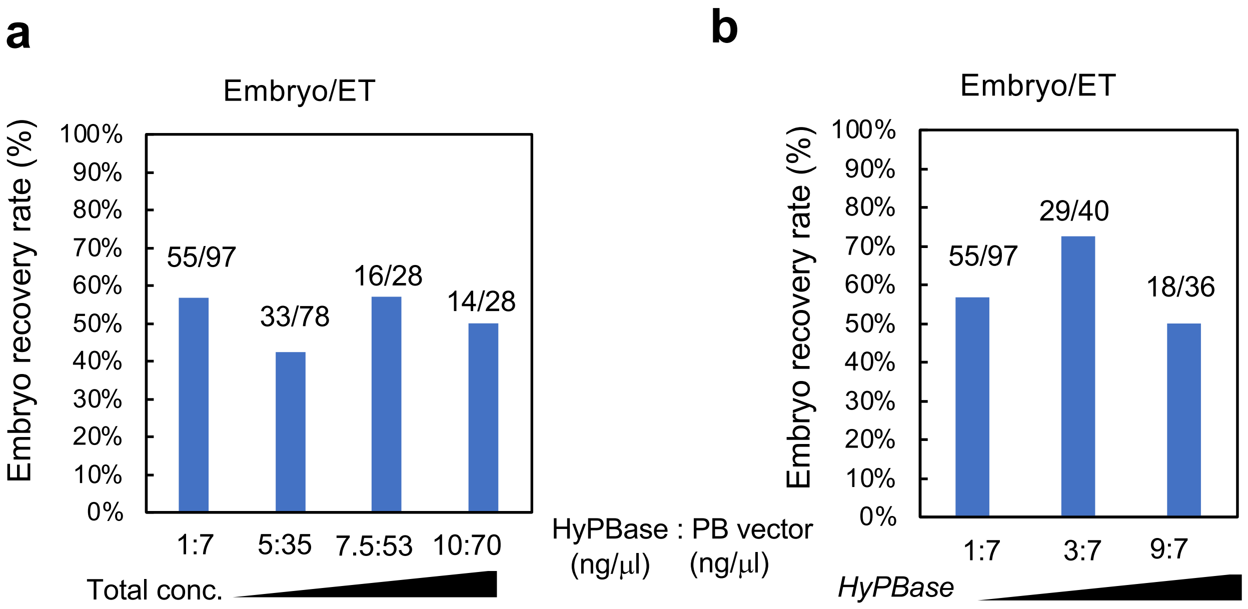

Fig. S3 Optimization of hyPBase and PB vector concentrations for generation of TG epigenome-edited mice. **(a)** Embryo recovery rate at 11.5 dpc under a constant concentration ratio of 1:7 hyPBase:PB vector. **(b)** Embryo recovery rate at 11.5 dpc under constant PB vector concentration (7 ng/µl).

a

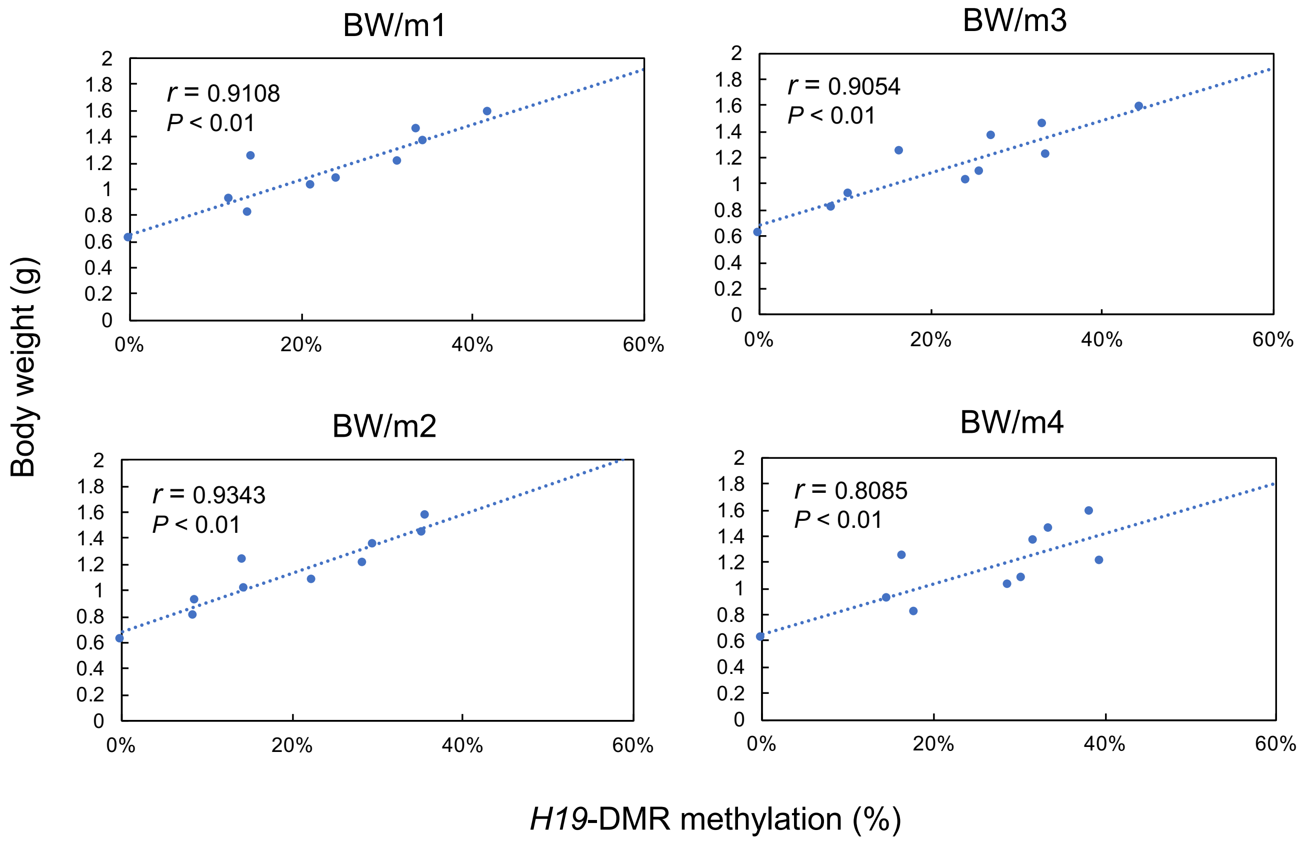

b

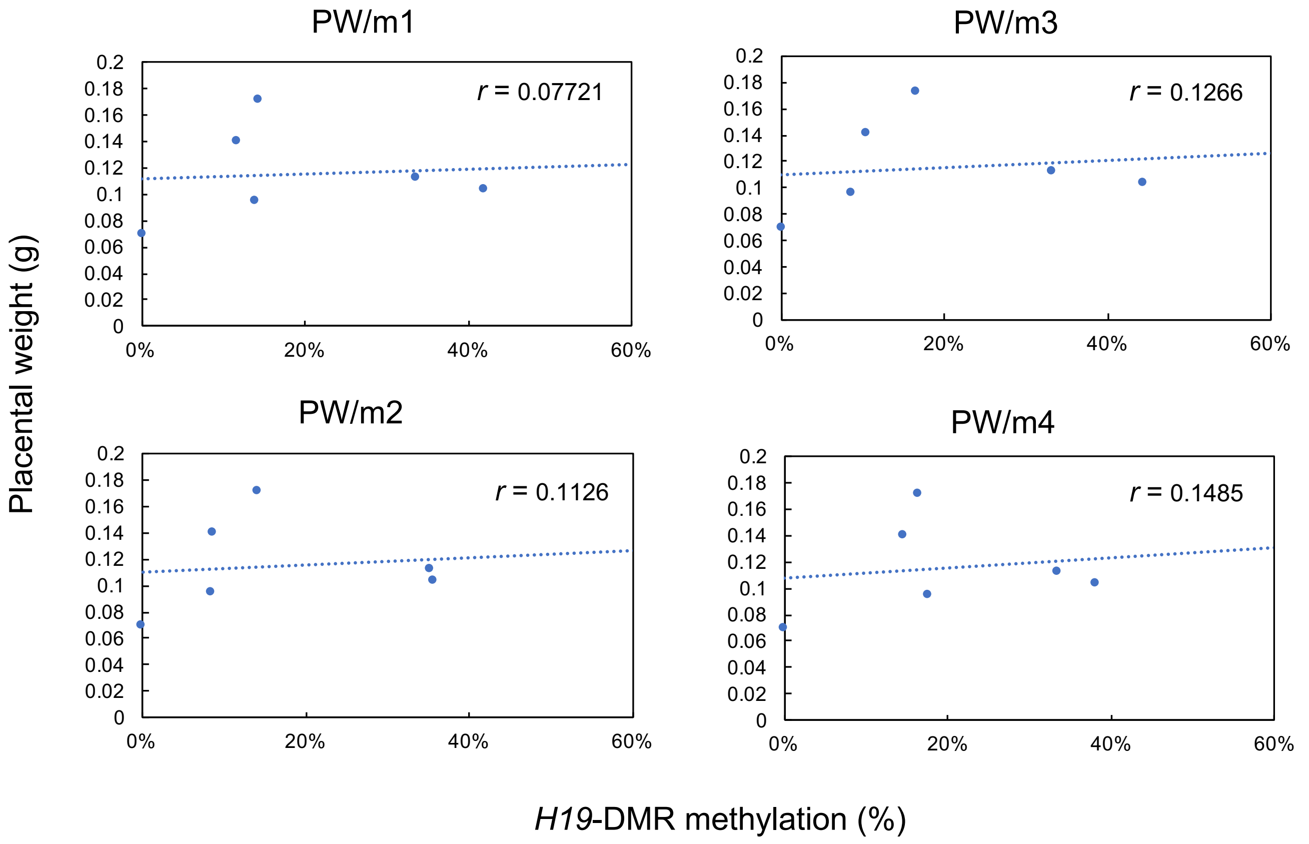

Fig. S4 Correlation of embryo DNA methylation with embryo and placental weights. **(a)** Strong correlation between *H19*-DMR DNA methylation at sites m1–m4 and body weight (BW) in TG epigenome-edited mice (n = 10). **(b)** Limited correlation between *H19*-DMR DNA methylation at sites m1–m4 and placental weight (PW) in TG epigenome-edited mice (n = 6). *r*, Pearson's correlation coefficient values.

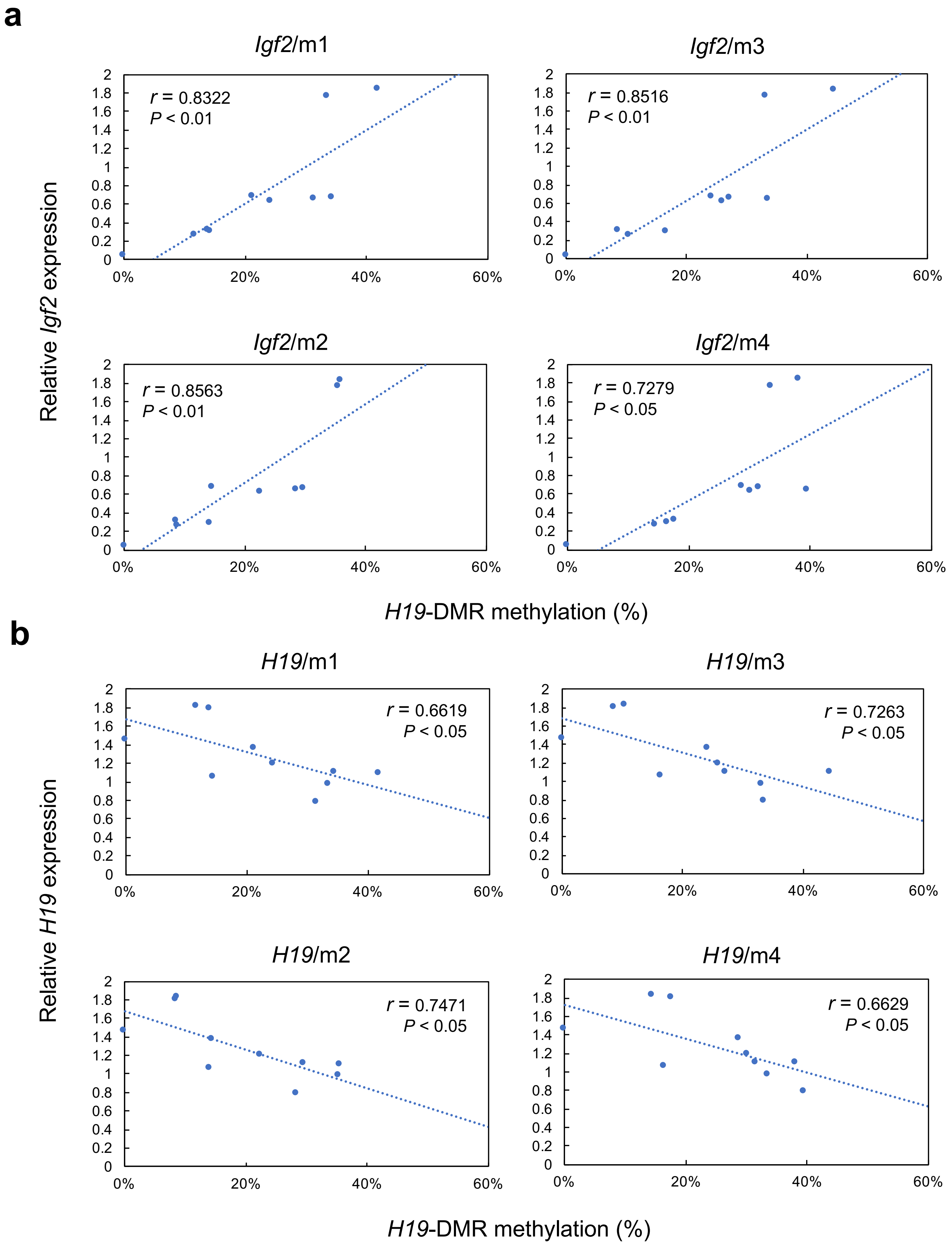

Fig. S5 Correlation between *H19*-DMR DNA methylation at sites m1–m4 and gene expression of **(a)** *Igf2* and **(b)** *H19* in TG epigenome-edited mice (n = 10). *r*, Pearson's correlation coefficient values.

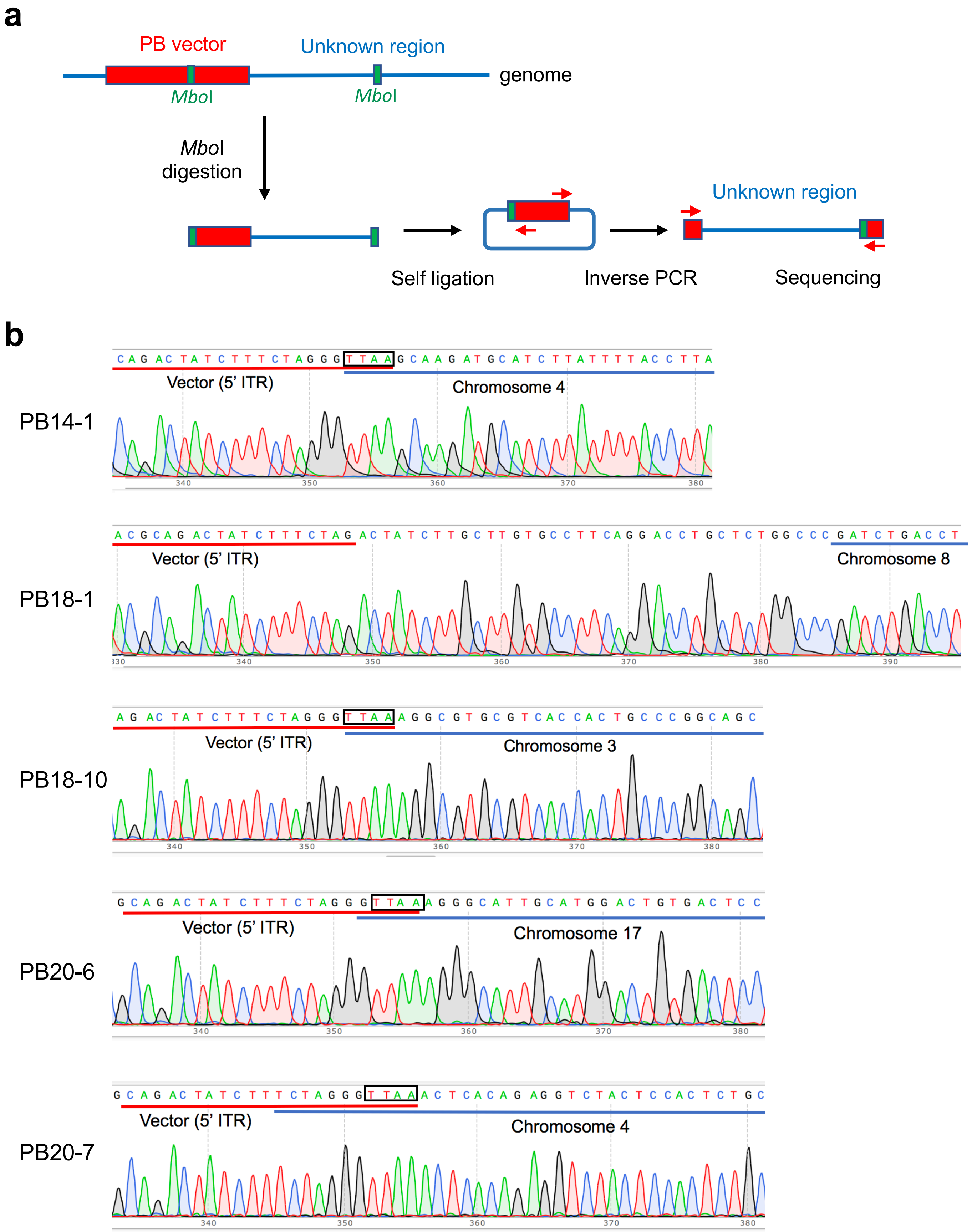

Fig. S6 Representative chromatograms of transgene integration loci.
(a) Schematics illustrating identification of transgene integration loci by self-ligation of genomic DNA digested with *Mbo*I followed by inverse PCR. **(b)** Except for PB18-1, TG mice showed PB vector integration at TTAA sites. By contrast, in PB18-1, 37 bases of filler DNA were detected between the vector and chromosome 8. In addition, six bases of the PB 5' ITR were deleted.

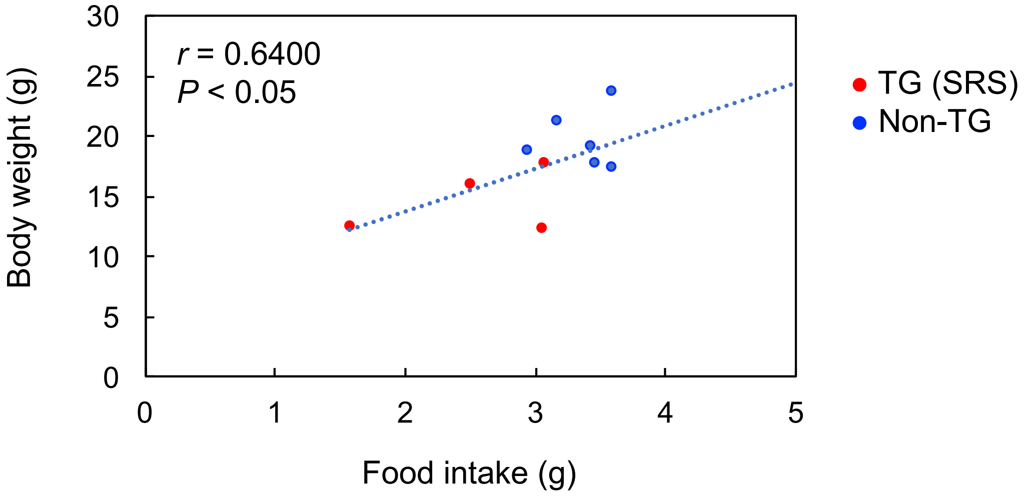

Fig. S7 Correlation between food intake and body weight.
A strong correlation was observed between food intake at 3 weeks and body weight at 5 weeks. This finding indicates that one reason why SRS mice remain small after birth is low food intake.
